# A Care Pathway Analysis of Tuberculosis Patients in Benin: Highlights on Direct Costs and Critical Stages for an Evidence-Based Decision-Making

**DOI:** 10.1371/journal.pone.0096912

**Published:** 2014-05-08

**Authors:** Samia Laokri, Arnaud Amoussouhui, Edgard M. Ouendo, Athanase Cossi Hounnankan, Séverin Anagonou, Martin Gninafon, Ferdinand Kassa, Léon Tawo, Bruno Dujardin

**Affiliations:** 1 Université Libre de Bruxelles, School of Public Health, Research centre Health Policy and Systems - International Health, Brussels, Belgium; 2 Institut Régional de Santé Publique, Unité de recherche, Ouidah, Benin; 3 Programme National contre la Tuberculose, Cotonou, Benin; University of California, San Francisco, United States of America

## Abstract

**Background:**

Free tuberculosis control fail to protect patients from substantial medical and non-medical expenditure, thus a greater degree of disaggregation of patient cost is needed to fully capture their context and inform policymaking.

**Methods:**

A retrospective cross-sectional study was conducted on a convenience sample of six health districts of Southern Benin. From August 2008 to February 2009, we recruited all smear-positive pulmonary tuberculosis patients treated under the national strategy in the selected districts. Direct out-of-pocket costs associated with tuberculosis, time delays, and care-seeking pattern were collected from symptom onset to end of treatment.

**Results:**

Population description and outcome data were reported for 245 patients of whom 153 completed their care pathway. For them, the median overall direct cost was USD 183 per patient. Payments to traditional healers, self-medication drugs, travel, and food expenditures contributed largely to this cost burden. Patient, provider, and treatment delays were also reported. Pre-diagnosis and intensive treatment stages were the most critical stages, with median expenditure of USD 43 per patient and accounting for 38% and 29% of the overall direct cost, respectively. However, financial barriers differed depending on whether the patient lived in urban or rural areas.

**Conclusions:**

This study delivers new evidence about bottlenecks encountered during the TB care pathway. Financial barriers to accessing the free-of-charge tuberculosis control strategy in Benin remain substantial for low-income households. Irregular time delays and hidden costs, often generated by multiple visits to various care providers, impair appropriate patient pathways. Particular attention should be paid to pre-diagnosis and intensive treatment. Cost assessment and combined targeted interventions embodied by a patient-centered approach on the specific critical stages would likely deliver better program outcomes.

## Introduction

With 8.7 million new cases worldwide in 2011, 1.4 million deaths, and 310,000 incident cases of multidrug-resistance, the management of tuberculosis (TB) remains a big challenge. [1] Sub-Saharan Africa has the highest rates of active TB per capita. [1] Nevertheless, in this region, Benin has the best results for TB control. [2] The national TB program (NTP) is well-established–one of the first supported by The Union and to implement the directly observed treatment, short-course (DOTS) strategy [3]–with detection and treatment success rates of 67% (2011) and 91% (2010), respectively, for new smear-positive patients. [4].

Despite these encouraging results, several bottlenecks persist such as financial barriers to access to care and too many treatment dropouts. [5] It is necessary to improve patients’ adherence to treatment. [6] Moreover, this disease disproportionally affects poor communities. While the TB control strategy is deemed cost-effective, at USD 100 per person successfully treated, [7] TB poses a severe burden on TB-affected households. Indeed, patient costs can be particularly burdensome in poor settings. [8].

Therefore, we closely studied the course of the long and complex care and control of TB suspects and patients. Our objectives were, from the user’s perspective, to describe the full range of disaggregated out-of-pocket direct costs (medical and non-medical) associated with tuberculosis, and to identify critical stages in order to determine any area for better patient management.

## Materials and Methods

### Ethics Statement

The study complies with international guidelines for research and was approved by the institutional review board of the Institut Régional de Santé Publique de Ouidah i.e., the Coordination de la recherche et des projets (BP 834 Ouidah, Benin). Informed consent was systematically requested. All subjects participating in the study signed a voluntary consent form after being given all the information necessary and sufficient to make an informed decision regarding their participation in this study.

### Study Setting

The study was conducted in six health districts of Southern Benin (i.e., Cotonou, Porto Novo, Come, Klouékanmé-Toviklin-Lalo, Pobé-Adja-Ouéré-Ketou, and Covè-Zagnanado-Ouinhi), covering a population of about 1.3 million people. Using convenience sampling method, the study sites were selected from Southern Benin that comprises health areas with the highest incidence rates for smear-positive TB cases (ranged from 24 to 59 per 100,000 population in Southern Benin versus only 13 to 14 per 100,000 population Northern Benin), highest density rates (ranged from 76 to 538 inhabitants per km^2^ versus only 30 to 35 inhabitants per km^2^), and for which we had an extensive knowledge of the healthcare networks and local communities [3], [4].

Since 1993, based on directly observed treatment, short-course (DOTS), the national tuberculosis control program (NTP) is fully integrated in health facilities. [9] All health centers are involved and responsible for the referral of TB suspects to the TB diagnostic and treatment centers (basic management units [BMUs] [10]). The BMUs cover the whole country, and the lab network is well decentralized. [3], [9] The NTP diagnostic algorithm requires repeated bacteriological examinations (i.e., three sputum samples collected within two days and microscopic examinations). Since April 2006, the treatment of smear-positive pulmonary TB cases is based on a six-month regimen. Drug intake is rigorously supervised (usually under hospitalization) during the two-month intensive treatment. [10] Before the continuation phase, a sputum control is performed. This four-month continuation treatment is self-administrated at home (through drug provided to the patient). [3] Two sputum controls are also done before declaring the patient cured. Under DOTS, TB diagnosis (based on sputum smear microscopy) and drug regimen are provided free of charge.

### Study Design and Participants

**Figure 1:**
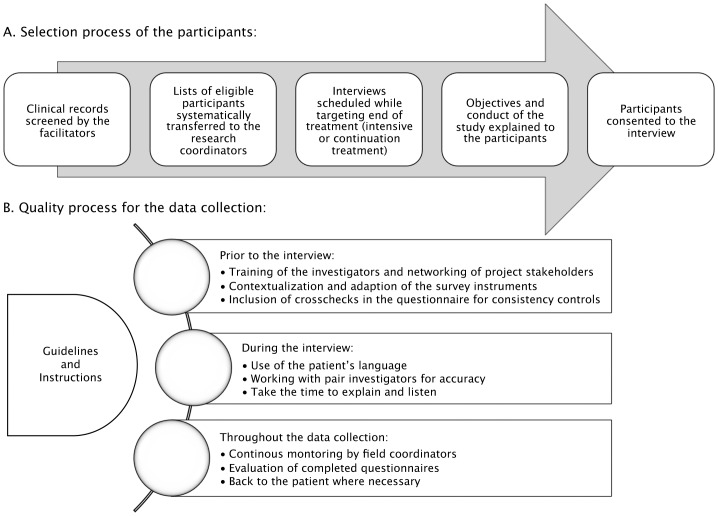
Participants’ selection and data collection process. Involving clinical heads to facilitate the selection process of the participants allowed to be comprehensive and to capture the target population. Similarly, several precautions were implemented at the different stages of the process to ensure best quality data.

Subsequent to our pilot study conducted in Burkina Faso, [11], [12] a retrospective cross-sectional study using a revised version of the original protocol was performed in Benin. The tool to estimate direct, indirect, intangible costs, and households strategies to cope with the costs associated with illness that we developed has been adapted to reflect the local context (Figure S1) [13]. In the selected districts, all smear-positive TB patients ≥15 years old enrolled in the NTP between August 2008 and February 2009 were recruited, including cured cases having completed their treatment in the last six months. Investigation was planned over a full year, but logistical constraints have led us to shorten the duration of the study. Both NTP managers and research team closely supervised research implementation. Figure 1 illustrates the selection process of the participants and data collection process. BMUs’ clinical heads facilitated the identification of eligible participants by screening clinical records. Among all patients attending NTP services, 250 patients met the inclusion criteria (i.e., adult ≥15 years old, smear-positive pulmonary TB patient, treated or cured under the NTP, and resident in the study sites) and were recruited. Then, we systematically scheduled interviews over the entire study period in order to target end of continuation treatment, or possibly end of intensive treatment for those who were still in treatment. Using a detailed and structured questionnaire, in-depth interviews were conducted by pair investigators the day of the control visits (or within a few days) for those in treatment or treated (i.e., the latest control visit of the stage), or later at home for cured cases. The questionnaire was pre-tested in July 2008. All patients were informed, consented and were interviewed in their own language for an average of two hours. Quality process prior and during the interview, and throughout the data collection process was implemented (Figure 1). When needed, an additional interview of the patient was performed by the same pair of investigators.

**Figure 2:**
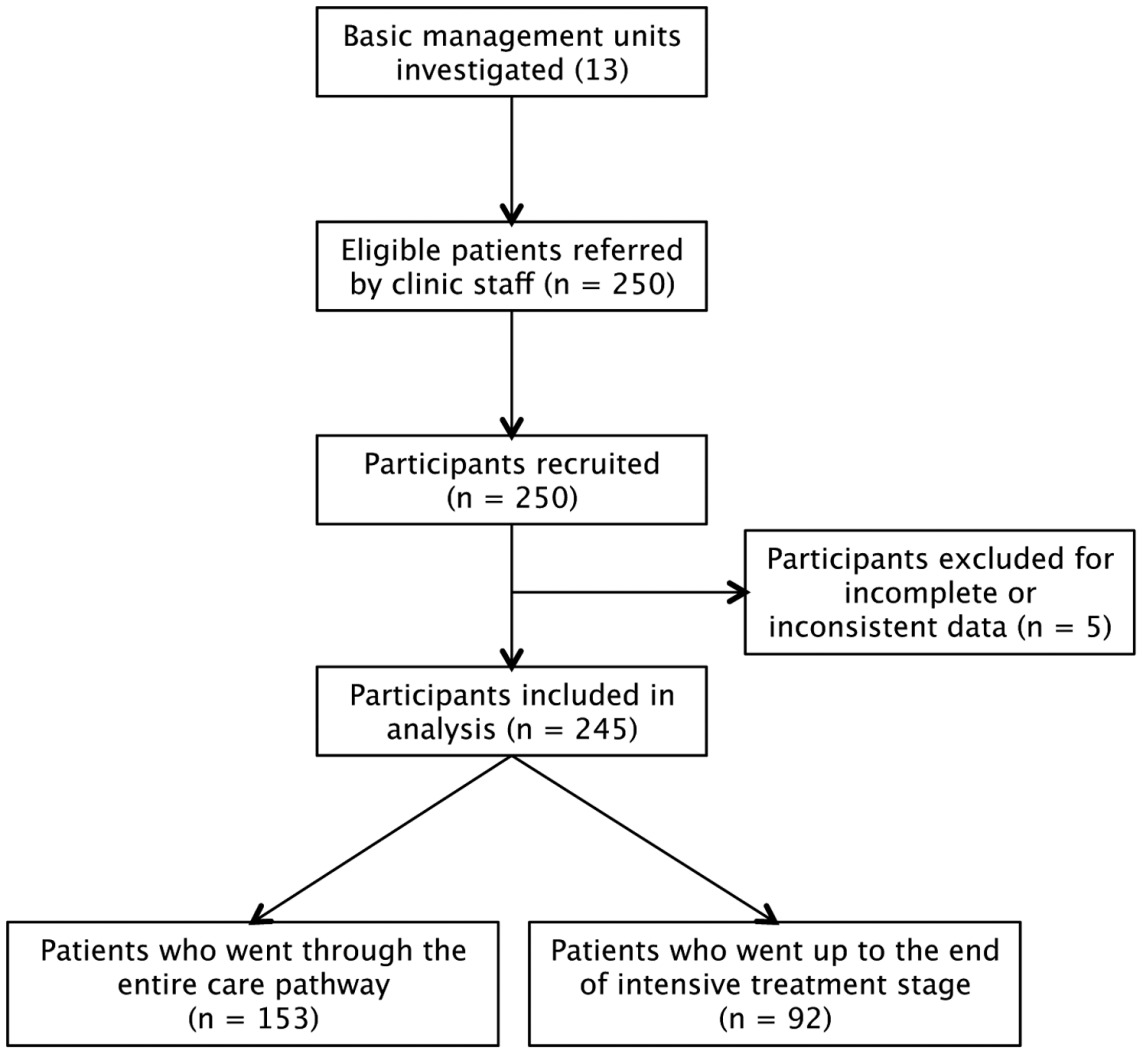
Flow diagram of the study participants. All participants (245/245) reported research outcomes from onset of their TB symptoms to intensive treatment while a smaller proportion of participants (153/245) reported research outcomes for their overall care pathway.

Due to the study design, enrollment rate was 100% (Figure 2). Out of the 250 participants enrolled, five were excluded for incomplete or inconsistent data. Two hundred and forty five (245) participants were included in the costing analysis. Two groups of participants consisted in 153 “treatment success” cases who completed treatment (142) or were cured (11), and 92 cases who were interviewed before starting continuation treatment. [14] The time since the patient had completed these respective stages to the time that the questionnaire was completed ranged from 0 days to a few days for 234 of the 245 participants (96%). For the remaining 11 participants that were cured (4%), this time period was less than a month for 9 participants and less than 3 months for 2 participants. Data related to the care pathway from onset of TB symptoms until the intensive treatment stage were reported by all patients while those related to the continuation treatment stage have been reported by a smaller proportion (62.4%) of patients.

### Data Processing

Itemized cost-of-tuberculosis assessment requested exhaustive statement of medical out-of-pocket expenses (e.g., the charges made for examinations, laboratory tests, drugs and hospital care, and consultation fees), and non-medical out-of-pocket expenses (e.g., services provided by traditional healers, traditional remedies, religious offerings, gifts for those who assisted in procuring and administrating care, food supplements or travel costs). By identifying their family events or other key dates, patients could relate the cost items in every successive stages of the TB care pathway. Expenses reported in-kind were converted into local currency during the interview. All cost items were summed to obtain the total cost, by stages and for the overall care pathway. Patients also commented on their socioeconomic status, care and care-seeking behaviors, time delays and major obstacles to access care. Conversion from the local currency to US dollars (USD) using OANDA Rates gave a mean price of USD 1 = 482.1 West African CFA Francs.

The Epi-Info (CDC, Atlanta, version 3·5·1) was used for data capture and cleaning. Data management and statistical analysis were processed with the IC/STATA 12 for Windows statistical package (StataCorp LP, USA). Continuous variables were described with means (and standard deviation) or medians (and interquartile values), and discrete variables with frequencies (and percentages). We operated the two-sample Wilcoxon rank-sum (Mann-Whitney) statistical test to compare distributions across sub-group categories of participants.

Study constraints occurred with pre-diagnosis stage in which 63 patients failed to recall the breakdown of some of their expenses for this particular period (especially food and transportation). Then they were asked to identify the types of expenses incurred and the corresponding total amounts. To preserve those data for analysis for those patients, we distributed linearly their expenses over the relevant cost items.

## Results

The average age of the participants was 35 years (SD = 13.2). The study included 146 males (59.6%). Of the sample, two thirds (66%) lived below the poverty threshold of USD 2.50 per person per day. The demographic and clinical patterns of the patients are presented in Table 1.

**Table 1:** Demographic and clinical pattern of the participants.

Category	Subcategory	Result (% (n))
Gender	Male	59.6 (146)
	Female	40.4 (99)
Age (missing = 1)	Age (Mean (SD))	35.0 (13.2)
Household size	<3	40.4 (99)
	[3]–[5]	33.9 (83)
	>5	25.7 (63)
Poverty	Living below US$ 1.25 per person per day	40 (98)
	Living above US$ 1.25 and below 2.50 per person per day	26 (64)
	Living above US$ 2.50 per person per day	44 (83)
Residence	Urban	64.5 (158)
	Rural	35.5 (87)
TB treatment category	New cases	91.8 (225)
	Retreatment cases	8.2 (20)
TB/HIV status (missing = 44)	Coinfected	15.7 (36)

### Overall Direct Cost

**Figure 3:**
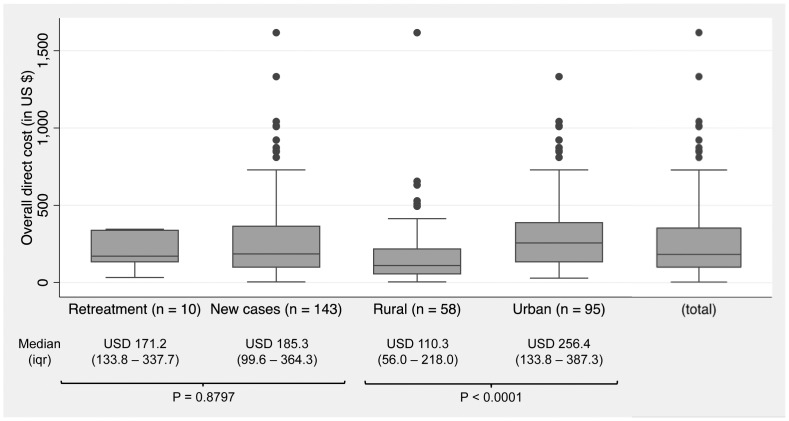
Overall direct cost of tuberculosis by category and region (in USD). Distributions of cost were widely spread. No statistical difference has been showed between new cases and retreatment cases (P = 0.8797). On the other hand, urban dwellers were substantially more likely to spend a higher overall direct cost than rural residents (P<0.0001).

Out of the 245 patients, overall median and interquartile out-of-pocket payments was USD 163.00 (USD 78.00–320.30) per patient. For the 153 “treatment success” cases, overall median out-of-pocket cost was slightly higher with USD 182.90 (USD 100.40–353.70) per patient. Figure 3 shows the wide dispersion of cost distributions as well as comparisons of different sub-groups of patients. New cases and retreatment cases had similar expenses. Urban dwellers were substantially more likely to spend a higher overall direct cost than rural residents (P<0.0001).

### Direct Cost and Care-seeking Pattern among the Patient Care Pathway

An analysis of the breakdown of the care pathway highlights the cost burden related to every stage of the TB patient’s pathway. Nature of payments, duration of stages, and care-seeking behaviors were specifically assessed for the successive stages (i.e., pre-diagnosis, diagnosis, treatment initiation, intensive treatment, and continuation treatment). The highest burden was likely in pre-diagnosis and intensive treatment, which can be qualified as the most critical stages of the patient care pathway (Table 2). While the total cost per stage is significantly higher among urban dwellers (except for diagnosis where rural residents spent more), we finally observe that the difference lies on two main expenditure items (self-medication, and travel and food costs). Beyond the magnitude of the overall cost per stage, Table 3 display the nature of expenditure incurred in every stage. The following paragraphs provide the occurrence and the extent of on these direct costs (Table 2 and Table 3) but also additional information such as care and care-seeking behaviours, time delays or main complaints regarding the successive stages.

**Table 2:** Direct out-of-pocket cost for tuberculosis in every stage of the patient care pathway (in USD).

Category	Participants*	Share of overalldirect cost acrossstages (%)**	Direct cost(in USD), All	Direct cost(in USD), Urban	Direct cost(in USD), Rural	Regionalcomparison
Stage of thecare pathway	n_1/_n_2_ (%)	Median (iqr)	Median (iqr)	Median (iqr, n)	Median (iqr, n)	P-value
Pre-diagnosis	228/245 (93.1)	38.2 (14.0–62.1)	43.0 (14.5–118.2)	62.2 (15.6–145.2, 149)	26.0 (10.4–67.4, 79)	0.0009
Diagnosis	241/245 (98.4)	12.3*** (5.7–33.1)	13.5 (9.7–34.6)	12.4 (9.7–20.2, 156)	22.8 (9.3–76.7, 85)	0.0077
Treatmentinitiation	184/245 (75.1)	1.5*** (0.5–3.8)	2.1 (1.0–4.1)	1.7 (1.0–3.7, 123)	2.1 (1.2–4.1, 61)	0.3905
Intensivetreatment	227/245 (92.7)	28.6*** (1.0–46.5)	43·4 (20.7–95.4)	55.6 (24.9–114.1, 153)	27.0 (12.4–55.5, 74)	<0.0001
Continuationtreatment	127/153 (83.0)	13.3 (2.4–28.5)	16.6 (4.1–68.5)	29.9 (6.2–95.8, 79)	11.1 (4.1–41.3, 48)	0.0226
Total	153/153 (100.0)	–	182.9 (100.4–353.7)	256.4 (133.8–387.3, 95)	110.3 (56.0–218.0, 58)	<0.0001

Median direct costs ranged from USD 2.10 per patient for treatment initiation to USD 43.40 per patient for intensive treatment. Pre-diagnosis and intensive treatment showed the highest median costs in both regions. Rural residents also incurred high burden during diagnosis stage.

*n1 = number of patients with direct cost >0 per stage; n2 = number of patients respectively who went through up to the intensive treatment stage (245), and who went through the entire care pathway (153).

**The median (iqr) share of overall direct cost across stages by region was: 44.0% (14.1–67.6) for pre-diagnosis, 8.5% (4.3–19.3) for diagnosis, 1.1% (0.4–2.9) for treatment initiation, 30.2% (16.2–49.8) for intensive treatment and 15.4% (2.0–29.5) for continuation treatment among urban residents, and respectively 32.1% (14.1–51.7), 27.3% (11.4–52.4), 2.2% (0.9–5.7), 22.2% (13.2–41.3) and 8.6% (3.4–22.7) among rural residents.

***Distributions of proportion significantly different across region (P<0.05).

**Table 3:** Major item costs >0 incurred in every stage of the patient care pathway (in USD).

Category	Sub-category	Occurrence	Direct cost(in USD), All	Direct cost(in USD), Urban	Direct cost(in USD), Rural	Regionaldifference
Stage of carepathway	Cost items	% of patients	Median (iqr, n)	Median (iqr, n)	Median (iqr, n)	P-value
Pre-diagnosis	Traditional healer	25.3	30.1 (10.5–83.0, 62)	31.1 (12.4–103.7, 39)	24.9 (4.1–51.9, 23)	0.1635
	Self-medicationand spiritual remedy	68.6	14.5 (7.2–41.5, 168)	20.74 (9.8–62.2, 100)	10,4 (6.2–20.7, 68)	0.0023
	Travel costs	59.2	10.4 (4.1–31.1, 145)	9.0 (3.3–24.9, 95)	11.0 (7.3–47.7, 50)	0.0523
Diagnosis	Medication	38.4	31.1 (10.4–77.3, 94)	31.1 (13.5–103.7, 38)	22.8 (7.8–57.0, 56)	0.1913
	Sputum-smear microscopy	62.0	1.0 (1.0–1.0, 152)	1.0 (1.0–1.0, 145)	1.2 (1.0–1.2, 7)	<0.0001
	Chest X-rays	60.4	6.2 (6.2–6.2, 148)	6.2 (6.2–6.2, 135)	10.4 (9.3–21.8, 13)	<0.0001
	Other medical costs(fees, additionalexamination)	78.4	2.1 (2.1–2.1, 192)	2.1 (2.1–2.1, 135)	1.5 (1.0–8.3, 57)	0.0858
	Travel costs	82.0	4.1 (2.1–10.4, 201)	3.4 (2.1–6.2, 121)	6.7 (3.7–20.7, 80)	<0.0001
Treatmentinitiation	All costs	75.1	2.1 (1.0–4.1, 184)	1.7 (1.0–3.7, 123)	2.1 (1.2–4.1, 61)	0.3905
Intensivetreatment	Medical costs	27.3	10.0 (3.7–20.7, 67)	10.0 (4.1–19.7, 53)	7.2 (3.1–20.7, 14)	0.7812
	Travel and food	89.0	41.5 (17.6–92.9, 218)	52.7 (20.7–112.0, 149)	31.1 (14.5–55.5, 69)	0.0008
Continuationtreatment	Medical costs	4.6	12.4 (7.3–20.5, 7)	12.4 (8.3–17.6, 5)	12.4 (4.3–20.4, 2)	–
	Travel and food	81.0	16.7 (4.1–66.9, 124)	31.1 (4.6–95.8, 77)	10.4 (3.1–38.6, 47)	0.0126

Most patients accumulated medical or non-medical out-of-pocket expenses at every single stage of their care pathways. The greater burdens relied on non-medical expenses during pre-diagnosis (traditional spending) and during intensive treatment (travel and food) while medical expenses were dominant during diagnosis stage. At the regional level, disparities between urban and rural residents were concentrated on the non-medical expenses.

#### Pre-diagnosis (patient delay)

From onset of symptoms to the first consultation (i.e., pre-diagnosis period, also referring to patient delay), 93.1% of the 245 patients were already facing direct costs associated with TB. The median burden amounted USD 43.00 per patient. A large majority of patients reported expenses for modern or traditional self-medication (68.6%) and travel (59.3%), and much less for traditional healers’ services (25.3%). However, the median expense incurred were USD 14.50 for self-medication, USD 10.40 for travel, and USD 30.10 for traditional healers. At the end, half of the patients had spent 38.2% of their overall direct costs during this stage. Cost-burden was more severe among urban dwellers. We can also say that out of the 244 (one missing data) individuals, less than one third (31.6%) consulted a public provider within a month. The patient delay in total ranged from less than a week to more than three weeks.

#### Diagnosis (provider delay)

Almost all the patients (98.4%) faced direct costs during the diagnosis period (i.e., from the first visit to diagnosis confirmation). The median burden was USD 13.50 per patient. Most patients reported expenses for travel (82.0%), consultation fees or additional examinations (78.4%), sputum microscopies (62.0%), or X-rays (60.4%), and the median expenditure for which amounted to USD 4.10, USD 2.10, USD 1.0, and USD 6.20 per patient, respectively. A smaller proportion of patients (38.4%) spent much higher amounts on medication (USD 31.10). This means half of the patients incur a cost of up to 12.3% of their overall direct cost. Cost-burden was much heavier among rural residents with a median of USD 22.80 and 27.3% of their overall direct cost. On the effectiveness of patient care, 55.3% of patients (135) were diagnosed with TB within a week, leaving a large number facing a provider delay longer than one week. Among all, 30.2% of the patients (74) resorted to private care before having met a public provider. Moderately, 131% (32) were hospitalized in order to diagnose TB with a median length of stay of three days (three to six). Among those who were not hospitalized, 11.5% of the patients (24) consulted public providers more than four times with a median of seven for care utilization (five to seven).

#### Treatment initiation (treatment delay)

Regarding treatment initiation, which refers to the period from announcement of the diagnosis to the start of treatment, 75.1% faced direct costs. The median burden was USD 2.10 per patient. It corresponded to a median share of overall direct cost of 1.5%. To the question “Have you faced a treatment delay,” 67.2% of patients spontaneously answered “yes.” After analysis of “travel to NTP providers,” it turns out that 84.9% of patients reported multiple (up to four) round trips. However, the mean treatment delay was two days (SD = 1.9).

#### Intensive treatment

Out of the 245 patients, 92.7% faced direct cost. The median burden was USD 43.40 per patient. Most patients (89.0%) faced median non-medical expenditures (travel or food) of USD 41.50. About a quarter (26.5%) faced median treatment expenditures (including drugs for minor illnesses) of USD 10.00 per patient. The median share of overall direct cost was 28.6%. In addition to this financial burden (reported by 26.6% of patients), other complaints were observed such as fatigue or suffering (30.8%), inactivity (11.1%), feeling of hunger or disturbance (9.4%), and poor access to BMUs or care providers (3%). Despite these challenges, less than one tenth discontinued their intensive treatment (6.9%; 17 patients). Some of the reasons for interrupting the drug regimen were as follows: one reported strike of health facilities, another ban on taking drugs invoked by the traditional healer; and the others reported occasional and personal inconveniences such as family event or inability to access the health center due to bad weather or vehicle breakdown. This stage lasted more than the two-month prescribed period for very few patients (2.6%).

#### Continuation treatment

Out of the 153 patients who went through the entire care pathway, 83.0% faced direct cost during continuation treatment. The median burden was USD 16.60 per patient for this four-month stage. A small majority of patients (50.6%) faced non-medical expenditures (travel or food), which achieved a median cost of USD 16.70. Only 2.9% faced treatment expenditures that amounted to a median cost of USD 12.40. Half of the patients spent 13.3% of their overall direct cost during this stage. On the performance of patient management, discontinuity in the drug regimen occurred for 6.6% of the patients. While financial barriers were reported by 27.8% of patients and regarded as principal obstacles even at this stage, other factors existed such as fatigue or suffering (22.5%), business interruption (11.3%), feeling of hunger (5.3%), geographic access (3.8%), or seclusion (0.8%).

## Discussion

The study showed very few households with no TB-related expenditure. The magnitude of these out-of-pocket payments reached a median of USD 256.40 per patient among urban residents and USD 110.30 per patient among rural residents, including medical and non-medical costs. In such disadvantaged socio-economic groups (i.e., high poverty rate reported), this amount may represent a substantial portion of their monthly income (or even exceed it) and thus weighs heavily on the household. From a user’s perspective, we showed several issues leading to genuine financial barriers. Overall financial burden complied with results already shown in the literature [8], [15] show that free TB control strategy comes at a very high cost. Our results also corroborate the findings from the situation analysis conducted in the project FORESA in Benin, [16] namely high costs associated with the management of TB (travel and food costs, follow-up costs, etc.), lack of information on the TB care circuit, and inefficiency of referral (low level of suspicion).

In a recent review of the literature covering 30 articles from sub-Saharan Africa, Barter et al [8] reported that total direct costs associated with TB widely ranged from USD 11 to USD 527, depending on the countries surveyed and cost items considered. Mean amounts spent in many countries are lower than the median amount spent in Benin (e.g. USD 59 in Tanzania, USD 49 in Ethiopia, USD 39 in Malawi, and USD 11 in Zambia). Only one study conducted in Ethiopia reported a value of USD 527. However, comparisons remain hazardous because expenses and pathways considered differ across studies. We particularly carried out a detailed comparison with Burkina Faso where our research protocol had been established in 2007. [11], [12] First, overall direct economic burden for the TB patients from rural Benin was fairly similar to the burden shown in rural Burkina Faso (median direct costs USD 110 vs. USD 101 per patient). The most critical stage for the households in rural Benin were pre-diagnosis (i.e., median cost-burden of 32% of the total out-of-pocket payments spent), diagnosis (27%) and intensive treatment phase (22%), whereas in rural Burkina Faso the most critical were the diagnosis (35%), treatment initiation (33%), and pre-diagnosis phase (24%). For urban residents in Benin, the most critical stages were pre-diagnosis, intensive treatment and to a lesser extent the continuation treatment. Nevertheless, differences in the severity of stages, and particularly among residents from urban and rural areas as shown in Benin, reinforces the need to focus on the contextual factors.

Countries should consider context-oriented policy initiatives that are evidence-based informed and guided by the patient needs. We raised some useful issues to promote such initiatives for TB control. During pre-diagnosis, one in four spent relatively large amounts on traditional healers in Benin. A third of the Beninese patients (32%) and two-thirds of the Burkinabe patients (57%) came to the first line healthcare provider and consulted in the first month of diagnosis. Likewise, delays worsened the burden of TB and contributed to potentially avoidable costs. [17] Shortening those critical periods, tackling behaviors of seeking care outside the formal care system and practicing self-medication remain a key issue in Benin. Sources of delays in Ethiopia [17] were inefficiencies in patients’ history of diagnosis (e.g., patients were not screened or diagnosed at the first visited diagnostic center), consultation with alternative providers, and transportation to seek care. Similar conditions existed in Benin, and these patient and treatment delays, although short, need to be tackled. From a TB control perspective, time delays observed in Benin exceeded ideal targets (i.e., no more than two or three weeks for patient delay and a few days for provider delay for a majority of smear-positive patients). [18] Appropriate testing should be requested and processed promptly for all suspects, [19] and supply-side interventions should be preferred. [18] When one third have not used the public care network or NTP provider as a first choice, the selection of provider for primary care seems like a milestone in the patient care pathway. [20] However, NTP strategy during diagnosis was relatively well followed but not within the two days necessary to provide the two sputum samples. Further evidence on the engagement of informal or private care providers in the national strategy would be of great interest. In addition, as in other policies such as maternal health, [21] another issue to emphasize is that expenses falling outside those supported by the NTP may have serious implications on the appropriateness of a care pathway and possibly may deter patients from public care providers. Indeed, policymakers need to discuss the relevance and the scope of certain expenses (e.g., x-ray or even care and drugs for diseases associated with TB such injections often reported by patients). Besides, some additional costs could become avoidable while improving patient management. We showed that a large majority of Beninese patients reported small spending on sputum smear-microscopy, which are supposed to be free-of-charge. Expenses for travel and food items were high among Beninese patients from urban areas throughout the intensive and continuation treatment phases. In line with findings from a systematic review in African countries, where treatment itself was free, patients reported various hidden costs that plagued the ideal pathway. [6] Furthermore, inappropriate pathways at significant costs can affect their micro-level socioeconomic position, and subsequently, further undermine the patient care pathway. [22].

### Study Limitations

Some study limitations can be reported. Firstly, a rural-urban setting was used to capture a wide variety of all the potential direct out-of-pocket costs associated with TB. Although costs are substantial throughout the sub-region, the severity of the problem tends to differ. In addition, costs were more widely spread among urban residents than among rural residents. Beyond the urban-rural gap, further studies should be conceived to grasp more local specificities. Secondly, to achieve accuracy and completeness, this type of study requires extreme caution. Quality of the data has been one of our primary concerns but recall bias should be considered and particularly for patients combining multiple health problems. A routine survey targeted on key issues could be very informative. Finally, further analyses such as to examine the relationship between the amount spent on out-of-pocket payments to household income, or documenting indirect costs and strategies to cope with direct costs are needed. Such analyses have not been reported here.

## Conclusions

The findings confirm that financial and organizational barriers to successful care seeking and treatment of TB–that also fall under the national “free” TB strategy–remain a major issue for TB-affected households. As argued by the authors of recent systematic reviews, evidence from our comprehensive pathway analysis answers the call for better documentation of the specific problems of African countries. A thorough, systematic study of patient choices may suggest evidence informed solution scenarios to improve their management. Critical stages regarding financial standpoints were likely pre-diagnosis, diagnosis (in rural area) and intensive treatment. Time delays (both patient and health system delays) and their cost implications contributed to the failure to suspect, diagnose, and treat TB early enough. Particular attention should also be paid to hidden costs that negatively affect the patient care pathway. Greater success of a long-course treatment relies on more integrated, context-oriented and patient-centered interventions, [23] and greater attention to access barriers. [6] Concerted and coordinated efforts among all stakeholders (i.e., other public health programs, [24] formal and informal care providers, community outreach, pharmacists, and patients) [25] and shared decisions about the management of health problems [6] will contribute to shorten both time delays and inappropriate patient management, and finally, lower overall burden. Policy process and contextualized implementation of the strategy to local needs and constraints, as we have seen these may vary across a same sub-region of Benin, are essential for realizing the full potential of the TB control policy. Finally, health policies would benefit from financial protection mechanisms for the targeted population and pre-payment and risk pooling strategies at country-level. The investment to implement the necessary interventions will have returns far exceeding costs, especially the positive impact on the patient that translates into household and community economic and social well-being.

## Supporting Information

Figure S1 **Tool to estimate the Economic Burden of Tuberculosis.** The tool intends to estimate the direct, indirect and intangible costs associated with tuberculosis in a user’s perspective. Disaggregated analysis enables to better understand the health-seeking behaviors and challenges across the various stages of the TB care pathway. The tool also comprises information on how patients mobilized financial resources to cope with direct costs, as well as social stigma and relationships.
(PDF)
