# Supplementary material for: A Care Pathway Analysis of Tuberculosis Patients in Benin: Highlights on Direct Costs and Critical Stages for an Evidence-Based Decision-Making
Source: PLoS One. 2014 May 8;9(5):e96912. doi: 10.1371/journal.pone.0096912 (PMC4014559; doi:10.1371/journal.pone.0096912)
Supplement: Figure S1 — Tool to estimate the Economic Burden of Tuberculosis. The tool intends to estimate the direct, indirect and intangible costs associated with tuberculosis in a user’s perspective. Disaggregated analysis enables to better understand the health-seeking behaviors and challenges across the various stages of the TB care pathway. The tool also comprises information on how patients mobilized financial resources to cope with direct costs, as well as social stigma and relationships. (PDF) [file pone.0096912.s001.pdf]

(In French below)

ULB ESP — BTC CTB EMPOWERING DEVELOPMENT — EUROPEAID OFFICE DE COOPÉRATION

F O R E S A

Formation Recherche-action Santé

www.ulb.ac.be/esp/psd/foresa

UNE INTERVENTION CONTINUÉE EN AFRIQUE DE L'OUEST

Un partenariat régional : Bénin, Burkina Faso, Côte d'Ivoire, Mali, Niger, Sénégal,
appuyé par l'Ecole de Santé Publique de l'Université Libre de Bruxelles, la Coopération Technique Belge
et financé par l'Union européenne (EuropAid contrat 2004 78-590)

SURVEY MATERIAL TO ESTIMATE COST OF TUBERCULOSIS IN RESOURCE-POOR COUNTRIES:
ASSESSING THE ECONOMIC BURDEN OF ILLNESS AND ITS CONSEQUENCES FOR THE PATIENT AND HOUSEHOLD

UNIVERSITE LIBRE DE BRUXELLES, APRIL 2007

Background: This survey is part of a broader research project, titled "*FOrmation continue, REcherche et action en Santé*", on tuberculosis control in sub-Saharan Africa. It has been initiated by the research unit Health Policy and Programs in Developing Countries of the School of Public Health of the Université Libre de Bruxelles in Belgium in close partnership with local partners in Benin and Burkina Faso. The EuropAid department of the European Commission (Projet Santé/2004 78-590) funded local staff and data collection.

Goal: To estimate direct, indirect and intangible patient costs associated with tuberculosis

Specific objectives

i. To exhaustively quantify medical and non-medical out-of-pocket payments incurred by tuberculosis-affected households over the entire care-seeking and care pathway
ii. To estimate the magnitude of and the risk associated with the economic burden of disease borne by tuberculosis-affected households
iii. To determine the extent of copying mechanims that may persist despite the free-of-charge diagnosis and treatment strategy
iv. To explore potential weaknesses and areas of progress of tuberculosis control strategies and contribute to inform policy-making

Contact of the author

Samia LAOKRI, Health economist
School of Public Health – Université Libre de Bruxelles
808, Route de Lennik – CP 594 – 1070 Brussels – Belgium
+32 (0)2 555 40 46 – slaokri@ulb.ac.be – www.ulb.ac.be/facs/esp/

Previous related publications:

•Laokri S, Weil O, Drabo MK, Dembélé SM, Kafando B, et al. (2013) Removal of user fees no guarantee of universal health coverage: observations from Burkina Faso. Bull World Health Organ: 277–282. doi:http://dx.doi.org/10.2471/BLT.12.110015.
•Laokri S, Drabo MK, Weil O, Kafando B, Dembélé SM, et al. (2013) Patients Are Paying Too Much for Tuberculosis: A Direct Cost-Burden Evaluation in Burkina Faso. PLoS ONE 8: e56752. doi:10.1371/journal.pone.0056752.

QUESTIONNAIRE D'ENQUETE POUR L'EVALUATION DE LA CHARGE ECONOMIQUE ASSOCIEE A LA TUBERCULOSE

ET SES CONSEQUENCES ECONOMIQUES ET SOCIALES POUR LE PATIENT ET SON MENAGE

UNIVERSITE LIBRE DE BRUXELLES, AVRIL 2007

INSTRUCTIONS PRELIMINAIRES :

Le FORMULAIRE D'INFORMATION ET DE CONSENTEMENT qui définit les objectifs et les conditions de participation à l'étude doit être présenté et explicité au participant afin que celui-ci puisse l'approuver avant le début de l'entrevue. Le présent questionnaire est constitué de sept parties à compléter soigneusement durant l'entrevue.

CONTENU :

INTRODUCTION DE L'ETUDE ET CONSENTEMENT DU PARTICIPANT 3
PARTIE 1. IDENTIFICATION DU PATIENT ET DE SON ENVIRONNEMENT 5
PARTIE 2. ITINERAIRE COMPLET DU PATIENT TUBERCULEUX (DEPUIS LES PREMIERS SYMPTOMES JUSQU'A L'ISSUE DU TRAITEMENT) 9
PARTIE 3. IDENTIFICATION DE L'AIDANT ET DE L'ACCOMPAGNANT AU MOMENT DE L'ENQUETE 13
PARTIE 4. PARCOURS DES SOINS ET COMPOSANTES DU COUT A CHARGE DU MENAGE 14
PARTIE 5. ESTIMATION DES DEPENSES TOTALES A CHARGE DU PATIENT 25
PARTIE 6. MODES DE PAIEMENT UTILISES POUR COUVRIR LES DEPENSES LIEES A LA TUBERCULOSE 26
PARTIE 7. COUTS INTANGIBLES 29

Introduction de l'étude et consentement du participant

INFORMATIONS SUR L'ETUDE. Vous allez participer à une étude sur l'évaluation des coûts directs, indirects et intangibles à charge du patient tuberculeux. Ce « Formulaire d'Information et de Consentement » contient des informations sur l'étude menée. Afin que vous soyez informé de tout ce que cette étude comporte, nous vous demandons de **lire attentivement (et/ou de vous faire lire et expliquer) ce qui suit**.

Les promoteurs de cette étude sont l'Ecole de santé publique de l'Université Libre de Bruxelles en Belgique, l'Institut Régional de Santé Publique de Ouidah au Bénin et l'Institut de Recherche en Sciences de la Santé de Ouagadougou au Burkina Faso. Ensemble, ils mènent actuellement des recherches sur les coûts de prise en charge des patients tuberculeux car ces coûts peuvent induire des dépenses importantes. De plus, ces coûts peuvent possiblement induire un certain déséquilibre dans la situation financière des ménages et contribuer ainsi à l'appauvrissement de la population. Des coûts importants peuvent également influencer significativement l'utilisation des services de santé. Les barrières financières constituent bien un frein important dans l'accès aux soins. Nous souhaitons mieux comprendre le contexte de votre parcours de soins. **Pour pouvoir participer à cette étude**, votre diagnostic tuberculose doit avoir été confirmé et vous devez être suivi par le programme national de lutte contre la tuberculose et pris en charge dans un des sites d'étude.

Objectif général de l'étude : La recherche s'attache particulièrement à l'estimation des coûts directs, indirects et intangibles relatifs à la prise en charge globale du patient tuberculeux et de sa famille dans le but de mettre en évidence d'éventuelles barrières financières et d'éclairer les décideurs politiques sur les points d'améliorations possibles en vue d'un meilleur fonctionnement des plans nationaux de lutte contre la tuberculose et de lutte contre la pauvreté.

Objectifs spécifiques de l'étude

1. Quantification des différents types de dépenses à charge du patient (et du ménage), en distinguant la nature des coûts et les parts relatives aux différentes phases de la maladie
2. Estimation de la charge économique et financière de la maladie pour le ménage
3. Identification d'une série de coûts potentiellement évitables
4. Identification des interventions réalistes à entreprendre, le cas échéant, pour diminuer le coût économique supporté par le ménage et formulation de recommandations en la matière (stratégies appropriées et pérennes pour mieux couvrir les dépenses nécessaires et améliorer l'accès aux services)

Quels sont les avantages attendus? Il est possible que vous ne retiriez aucun avantage de votre participation à cette étude. Si vous décidez de ne pas participer à cette étude, sachez que vous recevrez quoiqu'il en soit, les meilleurs soins habituellement dispensés pour votre maladie. Les informations et les résultats qui seront obtenus à l'issue de cette étude devraient permettre de mieux prendre en charge à l'avenir les patients tuberculeux.

Si vous acceptez de participer à cette étude : Votre rôle se limitera à l'administration du questionnaire. L'enquêteur vous posera de nombreuses questions, toutes sont listées dans ce document. Validez votre participation en signant la lettre de consentement (ou en y posant votre marque en présence d'un témoin) pour montrer que vous comprenez de quoi il s'agit. Nous vous invitons à nous poser autant de questions que nécessaire. Nous vous donnerons ensuite une copie datée et signée de ce formulaire d'information et de consentement. **Vos droits en tant que participant :** Cette étude a été revue et approuvée par un comité d'éthique indépendant. Un comité d'éthique indépendant est un groupe de personnes qui passe en revue les projets de recherche afin de s'assurer du caractère éthique des objectifs de recherche et de traitement des participants. Pour toutes questions sur vos droits en tant que participant(e) à l'étude, merci de contacter le comité scientifique de Coordination de la recherche et des projets (BP 834 Ouidah, Bénin). **Confidentialité.** Nous protègerons les informations concernant votre participation à cette étude. Votre nom n'apparaîtra dans aucun rapport. Si d'aventure nous vous contactons, nous ne divulguerons à personne votre participation à cette étude.

Abandonner l'Etude : La participation à cette étude est volontaire. Si vous décidez d'y participer, vous gardez cependant la possibilité d'abandonner l'étude à tout moment. En effet, si vous souhaitez arrêter l'entrevue, dites-le simplement à l'enquêteur.

LETTRE DE CONSENTEMENT A PARTICIPER A L'ETUDE

Je soussigné(e)____ certifie avoir parfaitement compris le contenu du présent formulaire et de la note d'information qui m'ont été présentés et commentés. J'ai eu l'opportunité de poser des questions et les réponses qui m'ont été données m'ont permis de comprendre davantage la nature et les objectifs de cette étude.

J'atteste avoir été informé sur les faits suivants:

1) Cette étude n'a pas d'intérêt direct pour moi. Cependant, il a un intérêt pour ceux qui luttent contre la tuberculose dans mon pays, car les informations que je donnerai ainsi que celles d'autres personnes qui participent à l'étude permettront de mieux orienter et prendre des décisions pour une meilleure approche de la prise en charge des patients tuberculeux.
2) Ma participation ne sera pas rémunérée. Cependant, si je me déplace, mon transport sera remboursé.
3) Les entretiens peuvent être enregistré sur bande audio. Cet enregistrement sera utilisé par l'équipe de recherche pour réécouter tous les éléments importants dits lors de l'entretien, éléments qui auraient pu échapper à la première écoute. A la fin de l'étude les enregistrements seront détruits.
4) L'entrevue reste anonyme et mon identité sera gardée secrète.
5) Je peux choisir de ne pas répondre à certaines ou à toutes le questions ou mettre fin à l'entretien n'importe quand.
6) Ma participation n'aura absolument aucun effet sur l'utilisation ou mes besoins de service de santé

Par ailleurs, j'accepte que les données recueillies à l'occasion de ce projet puissent faire l'objet d'un traitement informatique et de publications scientifiques. J'ai noté que mon droit d'accès aux données me concernant s'exerce à tout moment pendant la durée de l'étude et que je pourrais exercer mon droit de rectification et d'opposition pendant cette période.

Mon consentement ne décharge en rien les personnes réalisant l'étude de leurs responsabilités.

J'accepte librement de participer à cette étude selon les informations qui m'ont été données à partir de la note d'information et dans les conditions ci-dessus énoncées.

Fait à _____________, le___________/2007 Signature ou emprunte digitale du participant __________________

Je soussignée M __________________________________, enquêteur de l'étude, certifie avoir communiqué à M_________________________________ toutes les informations utiles sur cette étude. Je m'engage à faire respecter les termes de référence du présent formulaire de consentement et de réaliser la collecte des informations dans les conditions qui concilient le respect des droits et des libertés individuelles ainsi que le travail scientifique.

Fait à _____________, le___________/2007 Signature de l'enquêteur __________________

DATE DE L'ENTREVUE : …. /… / … LIEU : …………………… NOM DU BINOME ENQUETEURS : ……………………..

Partie 1. Identification du patient et de son environnement

1.1. N° de dossier du PNLT (identifiant) : …………………………… Date du diagnostic : …/…../…..

1.2. Zone sanitaire de …………………………… CdS/CdT de : ………...……./……………………

1.3. Nationalité : …………………………… Ethnie : ………………………………………………

1.4. Sexe : O Homme O Femme

Age : …… ans

Profession/Activité : O …………………………...O Sans profession

1.5. Statut matrimonial : O Marié(e), si oui précisez : O Monogame O Polygame et si épouse, précisez : Rang de l'épouse : …….

O Célibataire

O Veuf/Veuve

O Divorcé/Séparé

1.6. Composition de la maisonnée : Nbre d'enfants (<18 ans) : ………. Nbre d'autres habitants (>=18 ans): ……….

1.7. Chef de ménage : O Non, quel lien de parenté :……………………….. O Oui

1.8. Nombre de personnes à charge (famille élargie) : Nbre d'enfants (<18 ans) : …….. Nbre d'autres habitants (>=18 ans) : ……….

1.9. Type d'habitat : O maison en ciment O en banco O banco et ciment

Eau courante: O Non O Oui

1.10. Durée de résidence dans le quartier : ….. ans Précisez : O Locataire O Propriétaire

1.11. Quel(s) moyen(s) de locomotion possédez-vous au sein du ménage (plusieurs choix possibles) ?

O Charrette O Vélo O Pirogue O Moto O Voiture O Autre, précisez :………………………

1.12. Distance entre domicile et CdS (aller simple) : En Km :…… En temps: ….H ….min Coût moyen (1 personne) : ………….. FCFA

Distance entre domicile et CDT (aller simple) : En Km :…… En temps: ….H ….min Coût moyen (1 personne) : ………….. FCFA

1.13. Distance entre domicile et hôpital (aller simple) : En Km :…… En temps: ….H ….min Coût moyen (1 personne) : ………….. FCFA

1.14. Distance entre domicile et guérisseur (aller simple) : En Km :…… En temps: ….H ….min Coût moyen (1 personne) : ………….. FCFA

1.15. Quel moyen de transport est habituellement utilisé (trajets entre domicile, hôpital, CdS, CDT, guérisseur…) ?

O A pied O Charrette O Vélo O Transport fluvial O Moto O Voiture O Bus O Taxi O Autre, précisez ……………..

1.16. Niveau de scolarité : O Aucune O Alphabétisé (non formel) O Primaire O Secondaire O Supérieur

1.17. Statut Social, notoriété : O Très élevé O Elevé O Moyen O Faible O Isolé

1.18. Membre d'une organisation pour couvrir ses risques santé : O Non

O Oui, précisez: Nom de l'organisation : …………………

Montant de la cotisation? ……………….. CFA par ....

Couverture? ………………..

1.19. Estimez le revenu mensuel moyen du patient, avant sa maladie : ………….. FCFA /mois

1.20. a. Le patient est-il la seule source de revenu du ménage (toujours avant la maladie) ? O Non O Oui

1.20. b. Si oui, quelles autres sources de revenu :

1) Qui (statut par rapport au patient)? : ……………………… Quoi ? : ………………………Combien ? ……….………FCFA /mois

2) Qui (statut par rapport au patient)? : ……………………… Quoi ? : ………………………Combien ? ……….………FCFA /mois

3) Qui (statut par rapport au patient)? : ……………………… Quoi ? : ………………………Combien ? ……….………FCFA /mois

1.21. Estimez la variabilité des revenus totaux du ménage sur l'année? O Constants O Légèrement variables O Totalement variables

1.22. Identifiez les avoirs que possédait le ménage avant la maladie du patient ?

O Aucun avoir				
O Epargne	Quelle somme ?	………………… F CFA		
O Terre/Champs	Superficie ?	………………	Type de champs ?	………………
O Bétail	Nombre de bêtes ?	………………	Type de bêtes?	………………
O Réserve de vivre	Quantité de vivre?	………………	Type de vivre?	………………
O Autre possession, précisez	Quantité ?	………………	Type ?	………………

1.23. Où le patient se situe-t-il ? O Indigent O Pauvre O Moyen O Aisé

<u>CADRE STRICTEMENT RESERVE A L'ENQUETEUR:</u>

NOTE IMPORTANTE :

A COMPLETER <u>APRES L'ENTRETIEN</u>, EN CONSULTANT LE STATUT SEROLOGIQUE DU PATIENT DANS LE REGISTRE DE L'INSTITUTION DE SOINS (CDT) ?

1. 24. Statut coinfection TB/VIH du patient ?

- o TVNP: test VIH non proposé
- o TVPR: test VIH proposé mais refusé
- o TV+: test VIH positif (connu avant)
- o TV neg: test VIH négatif (connu avant)
- o TVP+: test VIH proposé et positif
- o TVP neg: test VIH proposé et négatif

Partie 2. Itinéraire complet du patient tuberculeux (depuis les premiers symptômes jusqu'à l'issue du traitement)

Figure 1 : Etapes clés du parcours de soins du patient TB

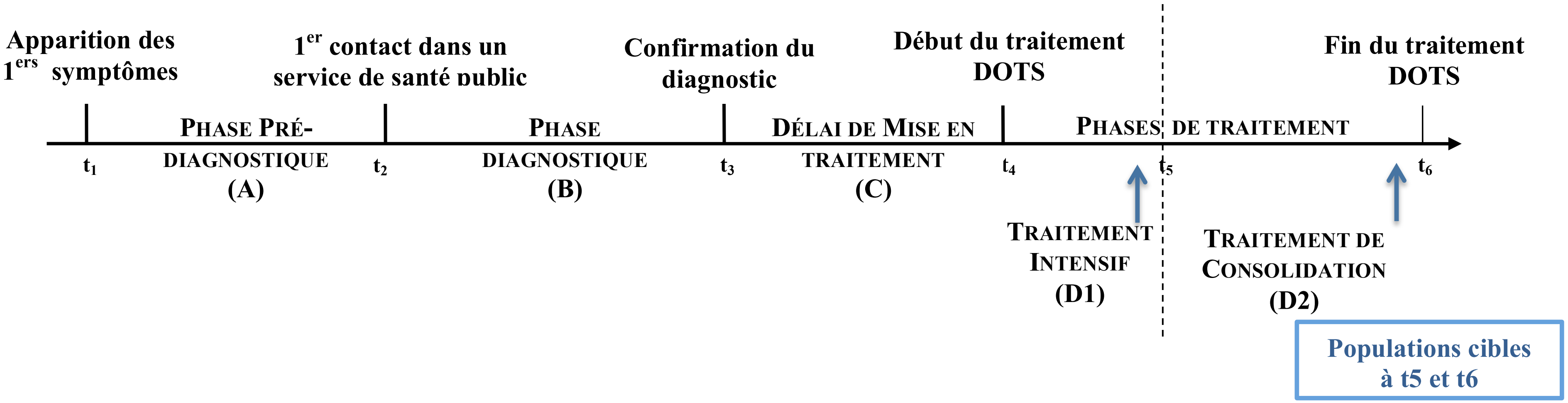

2.1. Quels symptômes initiaux (plusieurs choix possibles) ?

O Toux, précisez la période…….. ….. …………………

O Fièvre, précisez la période…….. ……………………

O Sang dans expectorations, précisez la période………………………

O Autre, précisez la période……………………………

2.2. Dans quelle phase de son itinéraire thérapeutique le patient se situe-t-il au moment de l'entrevue ?

O fin du traitement intensif

O fin du traitement de consolidation

O fin du retraitement intensif

O fin du retraitement de consolidation

O traitement terminé, le patient est guéri

2.3. Combien de temps s'est-il écoulé entre la découverte des **premiers symptômes discriminatifs** et le **premier contact** dans un service de santé (Phase pré-diagnostique – A) ?

………………………

O moins d'une semaine, précisez le nombre de jours : ……………..

O jusqu'à 1 semaine

O jusqu'à 2 semaines

O 3 à 4 semaines

O 1 à 3 mois

O plus de 3 mois

2.4. Combien de temps s'est-il écoulé entre le **premier contact** dans un centre de santé et l'annonce du **diagnostic** tuberculose au patient (Phase diagnostique – B) ? ……………..

O moins d'une semaine, précisez le nombre de jours : ……………..

O jusqu'à 1 semaine

O jusqu'à 2 semaines

O 3 à 4 semaines

O 1 à 3 mois

O plus de 3 mois

2.5. Combien de temps s'est-il écoulé entre l'annonce du **diagnostic** et le début du **traitement** (Phase de mise en traitement – C) ? …………………

O Zéro jour

O moins d'une semaine, précisez le nombre de jours : ……………..

O jusqu'à 1 semaine

O 2 semaines ou plus

2.6. Quelle est la durée du traitement intensif (Phase traitement intensif – D1) ? ………………………

O moins de 2 mois

O 2 mois

O plus de 2 mois

Note : Passez à la partie 3 si le patient est encore en phase de traitement intensif sinon, poursuivez avec la question suivante.

2.7. Quelle est la durée du traitement de consolidation (Phase traitement de consolidation – D2) ? ………………………

O moins de 4 mois

O 4 mois

O plus de 4 mois

Note : Passez à la partie 3 du questionnaire si le patient est encore en phase de traitement de consolidation sinon, poursuivez

2.8. Si le patient est en retraitement, combien de temps s'est-il écoulé entre la fin du **premier traitement** (traitement de première ligne) et le début du **second traitement** (traitement de seconde ligne) ? ………………………

O moins d'une semaine, précisez le nombre de jours : ……………..

O 1 semaine

O 2 semaines

O 3 à 4 semaines

O 1 à 2 mois

O plus de 2 mois

2.9. Si le patient est en retraitement, quelle est la durée du retraitement intensif ? ………………………

O moins de 2 mois

O 2 mois

O plus de 2 mois

Note : Passez à la partie 3 si le patient est encore en phase de retraitement intensif sinon, poursuivez avec la question suivante.

2.10. Quelle est la durée du retraitement de consolidation ? ………………………

O moins de 4 mois

O 4 mois

O plus de 4 mois

Partie 3. Identification de l'aidant et de l'accompagnant au moment de l'enquête

*Rappel : Il est important de distinguer les rôles de l'aidant et de l'accompagnant. L'**aidant** est la personne qui aide le patient à suivre son traitement ou encore qui le remplace pour assurer la continuité de ses activités (professionnelles). Et l'**accompagnant** est la personne qui accompagne le patient lors des ses différents déplacements pour soins (ex : visite au centre de santé, au CDT, ou à l'hôpital,…).*

3.1. Le patient a-t'il un aidant? O Non

O Oui, précisez ci-dessous

Est-il membre du ménage ?: O Non O Oui

Sexe : O M O F

Age : …… ans

Profession/Activité de l'aidant: O …………………………... O Sans profession

Qui est l'aidant (c.à.d. son statut par rapport au patient)? : ……………………………………………

3.2. Le patient a-t'il un accompagnant? O Non

O Oui, il s'agit de l'aidant

O Oui, il s'agit d'une autre personne, précisez ci-dessous

Est-il membre du ménage ?: O Non O Oui

Sexe : O M O F

Age : …… ans

Profession/Activité de l'aidant: O …………………………... O Sans profession

Qui est l'accompagnant (c.à.d. son statut par rapport au patient)? : ……………………………………………

Partie 4. Parcours des soins et composantes du coût à charge du ménage

Cette partie du questionnaire porte sur des ***questions spécifiques à chacune des phases*** *de l'itinéraire diagnostique et thérapeutique du patient (pour mémoire, voir figure 1 présentée au début de la partie 2). Aidez le patient à situer les moments clés au moyen de repères qui leurs sont familiers. Ces sous-parties se composent d'une série de questions et d'un tableau destiné à identifier l'ensemble des coûts directs (c.à.d. les dépenses) à charge du ménage et des coûts indirects (càd les journées perdues) et doivent strictement correspondre à la phase mentionnée. Pour chaque phase, vous avez quelques questions d'introduction et puis un TABLEAU à compléter soigneusement. Dans ce tableau :* ***Listez toutes les dépenses médicales et non médicales*** *faites pour la tuberculose, rapportez également l'explication en toute lettre. Précisez également le nombre précis de* ***journées de perdues à cause de la tuberculose*** *dans le but d'estimer le temps de travail perdu.*

Phase A - Sous-partie concernant la phase pré-diagnostique

4.1. Qu'a fait le patient après l'apparition des premiers symptômes et avant de consulter dans un service de santé (plusieurs choix possibles) ?

O Rien

O Auto-traitement

O Recours au guérisseur, nombre de contacts :…… fois

4.2. Le patient est-il assisté durant cette phase pré-diagnostique – A ? O Non O Oui

4.3. Si oui, veuillez préciser le type d'assistance (plusieurs choix possibles):

O Aidant. Discuté avec guérisseur ? : O Non O Oui

O Accompagnant

O Soutien affectif, précisez qui/quoi/combien ? ………………………………………………………………………………………

O Soutien financier, précisez qui/quoi/combien ? ……………………………………………………………………………………

O Autre type d'aide, qui/quoi/combien ? : ……………………………………………………………………………………

4.4. Tableau des dépenses du ménage réalisées pendant la phase pré-diagnostique – A

	MONTANTS EN Francs CFA	A QUOI CORRESPONDENT CES DEPENSES ?
1. Frais chez le guérisseur :<br>Avec paiement en argent		
Avec paiement en nature		
2. Automédication :<br>Avec paiement en argent		
Avec paiement en nature		
3. Déplacement (transport et alimentation)		
4. Autre dépense, veuillez préciser :		

	JOURS PERDUS	A QUOI CORRESPONDENT CES JOURS ?
5. Par le patient :<br>à cause de son invalidité		
à cause des déplacements réalisés		
6. Par l'aidant :<br>pour le soutien du patient		
pour remplacement du patient		
7. *Par l'accompagnant*<br>pour les déplacements réalisés avec ou à la place du patient		

Phase B - Sous-partie concernant la phase diagnostique

4.6. Quand avez-vous consulté pour la première fois un service public de santé : ……. /……... /……... (jour/mois/année)

Lequel ? : ……………………………….

Le patient a été envoyé par : O le guérisseur

O un professionnel de la santé

O le chef de famille

O autre : …………………….

Premier contact ? O service public

O service privé

4.7. Combien de contacts auprès de prestataires modernes avez-vous eu avant l'annonce du diagnostique ? ……. fois

4.8. Avez-vous été hospitalisé ? O Non O Oui, combien de jours ? ………….. jours

4.9. Le patient est-il assisté durant cette phase B ? O Non O Oui

4.10. Si oui, veuillez préciser le type d'assistance (plusieurs choix possibles):

O Aidant. Discuté avec prestataire ? O Non O Oui

O Accompagnant

O Soutien affectif, qui/quoi/combien ? ………………………………………………………………………………………

O Soutien financier, qui/quoi/combien ? ………………………………………………………………………………………

O Autre type d'aide, qui/quoi/combien ? ………………………………………………………………………………………

4.11. Tableau des dépenses du ménage réalisées pendant la phase diagnostique (Phase B)

	MONTANTS EN Francs CFA	A QUOI CORRESPONDENT CES DEPENSES ?
1. Honoraires de consultations		
2. Hospitalisation		
3. Traitement et soins		
4. Examens médicaux :<br>Expectoration (incl. test sputum)		
Radiologie (incl. rayon x poumon)		
Examens complémentaires		
5. Déplacement (transport et alimentation)		
6. Autre, veuillez préciser		

	JOURS PERDUS (phase B)	A QUOI CORRESPONDENT CES JOURS ?
7. Par le patient :<br><br>à cause de son invalidité		
à cause des déplacements réalisés		
8. Par l'aidant :<br><br>pour le soutien du patient		
pour les remplacement du patient		
9. Par l'accompagnant<br><br>pour les déplacements réalisés avec ou à la place du patient		

4.12. Quelle est la principale difficulté que le patient a rencontrée durant la phase diagnostique (phase B) ? :

………

………

………

………

……

Phase C - Sous-partie concernant la phase de mise en traitement, c.à.d. avant le début du traitement et après l'annonce du diagnostic

4.13. Y a-t-il eu un délai (décalage de temps) entre l'annonce du diagnostic et le début traitement: O Non

O Oui, nbre de jours:…………………………

Combien de fois vous êtes-vous déplacé vers un service public de santé pendant cette phase de mise en traitement : …… fois

Estimez le total de vos dépenses associées à la TB pour cette période :……………………….. CFA

Commentaire libre : ……………………………………………………………………………………

……………………………………………………………………………………

……………………………………………………………………………………

Phase D1 - Sous-partie concernant la phase de traitement ou de retraitement intensif

4.14. Le traitement (ou le retraitement intensif) a-t-il dû être interrompu à ce stade ? O Non

O Oui, pourquoi ? ………………………………………………………………

4.15. Le patient est-il assisté durant cette phase intensive ? O Non O Oui

4.16. Si oui, veuillez préciser le type d'assistance (plusieurs choix possibles):

O Aidant. Discuté avec prestataire ? : O Non O Oui

O Accompagnant

O Soutien affectif, qui/quoi/combien ? ……………………………………………………………………………………

O Soutien financier, qui/quoi/combien ? ……………………………………………………………………………………

O Autre type d'aide, qui/quoi/combien ? ……………………………………………………………………………………

4.17. Tableau des dépenses du ménage réalisées pendant la phase intensive de traitement (ou de retraitement intensif)

	MONTANTS en Francs CFA	A QUOI CORRESPONDENT CES DEPENSES ?
1. Honoraires médicaux		
2. Hospitalisation		
3. Traitement et soins (hors hospitalisation)		
4. Examens médicaux:<br>Expectoration (incl. test sputum)		
Radiologie (incl. rayon x poumon)		
Examens complémentaires		
5. Régime alimentaire ou boisson spécifique		
6. Déplacement (transport et alim.)		
7. Autre, veuillez préciser :<br>………………………………………		

	JOURS PERDUS (pdt le traitement intensif)	A QUOI CORRESPONDENT CES JOURS ?
8. Par le patient :<br>à cause de son invalidité		
à cause des déplacements réalisés		
9. Par l'aidant :<br>pour le soutien du patient		
pour le remplacement du patient		
10. Par l'accompagnant :<br>pour les déplacements réalisés avec ou à la place du patient		

4.18. Quelle est la principale difficulté que le patient a rencontrée durant la phase intensive de traitement ou de retraitement ?

………

………

………

………

………

Phase D2 - Sous-partie concernant la phase de traitement ou de retraitement de consolidation

Note : Si le patient n'a pas terminé la phase intensive de son traitement ou de son retraitement, alors passez directement à la partie 5 du questionnaire.

4.20. Le traitement ou le retraitement de consolidation a-t-il dû être interrompu ? O Non O Oui, pourquoi ?……………………..……………………..

4.21. Le patient est-il assisté durant cette phase de consolidation ? O Non O Oui

4.22. Si oui, veuillez préciser le type d'assistance (plusieurs choix possibles):

O Aidant. Discuté avec prestataire ? O Non O Oui

O Accompagnant

O Soutien affectif, qui/quoi/combien ? ....

O Soutien financier, qui/quoi/combien ? ....

O Autre type d'aide, qui/quoi/combien ? ....

4.23. Tableau des dépenses du ménage réalisées pendant la phase de traitement de consolidation

	**MONTANTS en Francs CFA**	**A QUOI CORRESPONDENT CES DEPENSES ?**
1. Honoraires médicaux		
2. Hospitalisation		
3. Traitement et soins (hors hospitalisation)		
4. Examens médicaux :<br>Expectoration (incl. test sputum)		
Radiologie (incl. rayon x poumon)		
Examens complémentaires		
5. Régime alimentaire ou boisson spécifique		
6. Déplacement (transport et alimentation)		
7. Autre, veuillez préciser		

	JOURS PERDUS (phase de consolidation)	A QUOI CORRESPONDENT CES JOURS ?
8. Par le patient :<br><br>à cause de son invalidité		
à cause des déplacements réalisés		
9. Par le l'aidant :<br><br>pour le soutien du patient		
pour le remplacement du patient		
10. Par l'accompagnant<br><br>pour les déplacements réalisés avec ou à la<br><br>place du patient		

4.24. Quelle est la principale difficulté que le patient a rencontrée durant la phase de traitement ou de retraitement de consolidation ?

………

………

………

………

………

Partie 5. Estimation des dépenses totales à charge du patient

5.1. Estimez le montant total des dépenses réalisées à ce jour par le patient pour la prise en charge de sa maladie: ………………………….. FCFA

ESPACE POUR COMMENTAIRES LIBRES

………

………

………

………

………

………

………

………

………

………

………

………

………

………

Partie 6. Modes de paiement utilisés pour couvrir les dépenses liées à la tuberculose

Cette partie du questionnaire vise à mieux comprendre comment le patient et son ménage ont payé toutes les dépenses de la tuberculose relevées jusqu'ici.

6.1. Utilisation de l'épargne du ménage ? O Non O Oui, précisez …………..FCFA Quelle épargne ?..

6.2. Mise en gage de biens précieux appartenant au ménage (bijoux et parures, …) ? O Non O Oui, précisez …………..FCFA Quels biens?.......................................

6.3. Restrictions de la consommation ? O Non O Oui, précisez ……………..FCFA O Biens non essentiels? ...………… O Biens essentiels?……………

6.4. Vente des avoirs appartenant au ménage (plusieurs réponses possibles) ? O Non O Oui, précisez ci-dessous

O	réserve de vivre /stocks alimentaires	……………….. FCFA	Quoi ?...
O	bétail	……………….. FCFA	Quoi ?...
O	champs (superficie)	……………….. FCFA	Quoi ?...
O	habitation	……………….. FCFA	Quoi ?...
O	biens précieux (bijoux et parures)	……………….. FCFA	Quoi ?...
O	moyens de locomotion	……………….. FCFA	Quoi ?...
O	autre	……………….. FCFA	Quoi ? ....

6.5. Vente des avoirs n'appartenant pas au ménage (plusieurs réponses possibles) ? O Non O Oui, précisez ci-dessous

O	réserve de vivre /stocks alimentaires	……………….. FCFA	Quoi ?...
O	bétail	……………….. FCFA	Quoi ?...
O	champs (superficie)	……………….. FCFA	Quoi ?...
O	habitation	……………….. FCFA	Quoi ?...
O	biens précieux (bijoux et parures)	……………….. FCFA	Quoi ?...
O	moyens de locomotion	……………….. FCFA	Quoi ?...

O autre ……………….. FCFA Quoi ? ....

6.6. Emprunt ou Endettement ?

6.6.1. au sein du ménage (ou famille élargie) : O Non O Oui, précisez ci-dessous

Qui ? ....

Quoi ? ....

Combien ? …....................…………. FCFA

6.6.2. en dehors du ménage (réseau social) : O Non O Oui, précisez ci-dessous

Qui ? ....

Quoi ? ....

Combien ? …...................………….. FCFA

6.7. Services ayant été demandé à quelqu'un d'extérieur au ménage : O Non O Oui, précisez ci-dessous

Quel service? ....

Montant: ....FCFA

Qui a rendu le service? ....

Quel type contrat/arrangement entre les parties? ....

6.8. Services prestés par le patient ou un autre membre du ménage: O Non O Oui, précisez ci-dessous

Quel service? ....

Montant: ....FCFA

Qui a rendu le service? ....

Quel type contrat/arrangement entre les parties? ....

6.9 Perte de revenus liée à la suspension ou l'arrêt des activités du patient, de l'aidant et de l'accompagnant : O Non O Oui, précisez ci-dessous

Quoi? ....

Montant:..FCFA

6.10 Réduction des investissements du ménages (retrait de l'enfant de l'école, ….) O Non O Oui, précisez ci-dessous

Quoi? ....

Montant:..FCFA

6.11 Autres stratégies développées par le patient ou le ménage pour faire face aux coûts de la tuberculose (coûts directs ou indirects) ? Précisez ci-dessous

……………………………………………………………………………………………………… ……………………………. FCFA

……………………………………………………………………………………………………… ……………………………. FCFA

……………………………………………………………………………………………………… ……………………………. FCFA

Partie 7. Coûts intangibles

Sous-Partie (a). Stigmatisation sociale

7.1.1. Qu'est-ce que la tuberculose a le plus changé pour vous ? Quels problèmes sociaux avez-vous perçus ?

O Isolement (au sein famille). Expliquez et depuis quand ? ……………………………..………………………..…………............

………

O Rejet social (au sein de la communauté). Expliquez et depuis quand ? ……………………………..……………………...…………

………

O Autre:……………………………………. Expliquez et depuis quand ? ……………………………..……………………...…………

………

7.1.2. Avez-vous ou devez-vous changer d'activité professionnelle à cause de la TB ? O Non O Oui, précisez ci-dessous

Quelle est la raison ? ……………………………………………………………………………………………

Depuis quand ? ou pour quand ? ……………………………………………………………………………………………

Quel changement ? …………………………………………………………………………………………

7.1.2.1.1. Votre rôle et votre position dans le ménage ont-ils changé ? O Non O Oui, précisez ci-dessous

Quelle est la raison ? ……………………………………………………………………………………………

Depuis quand ? ou pour quand ? ……………………………………………………………………………………………

Quel changement ? ……………………………………………………………………………………………

7.1.4. Quelle a été votre réaction vis-à-vis de l'ensemble des problèmes rencontrés ?

O J'ai pu surmonter le problème sans difficulté

O J'ai pu surmonter le problème mais avec difficultés. Expliquez ……………………….………………………..………………..

……

O Je n'ai pas pu surmonter le problème.   Expliquez ………………………..…………………..……………………………….

……

Sous-Partie (b). Dette Sociale

7.1.3. 7.2.1. Le patient se sent-il redevable **envers ses proches / sa famille** ? O Non O Oui, précisez ci-dessous

De quoi est-il redevable? ....……………………………

Qu'est-ce que le patient devra faire pour en être quitte ?..

7.1.4. 7.2.2. Le patient et son ménage se sentent-ils redevables **envers la communauté** (réseau social) ? O Non O Oui, précisez ci-dessous

De quoi est-il redevable? ....……………………………

Qu'est-ce que le patient devra faire pour en être quitte ?..

Fin. Nous vous remercions de votre participation.
